# Predicting Cervical Cancer Outcomes: Statistics, Images, and Machine Learning

**DOI:** 10.3389/frai.2021.627369

**Published:** 2021-06-07

**Authors:** Wei Luo

**Affiliations:** Department of Radiation Medicine, University of Kentucky, Lexington, KY, United States

**Keywords:** cervical cancer, clinical outcome prediction, statistical model, machine learning, medical image, radiomics

## Abstract

Cervical cancer is a very common and severe disease in women worldwide. Accurate prediction of its clinical outcomes will help adjust or optimize the treatment of cervical cancer and benefit the patients. Statistical models, various types of medical images, and machine learning have been used for outcome prediction and obtained promising results. Compared to conventional statistical models, machine learning has demonstrated advantages in dealing with the complexity in large-scale data and discovering prognostic factors. It has great potential in clinical application and improving cervical cancer management. However, the limitations of prediction studies and prediction models including simplification, insufficient data, overfitting and lack of interpretability, indicate that more work is needed to make clinical outcome prediction more accurate, more reliable, and more practical for clinical use.

## Introduction

Cancer treatment is one of the most complicated and challenging tasks in medicine. Although cancer survival rate has been significantly improved for the last decades with the introduction of new drugs, technologies and techniques, there are still uncertainties on the effect of those advances on clinical outcomes. The information of clinical outcomes is critical for the evaluation of treatment effectiveness and optimization of treatment strategies. Clinical outcomes usually are not available until enough clinical data have been accumulated following up a large number of patients for long periods. To know clinical outcomes more quickly so that treatment can be improved or adjusted timely, accurate prediction of clinical outcomes is expected. There are two approaches used for clinical outcome prediction. One is to use radiobiological models including tumor control probability (TCP) model, normal tissue complication probability (NTCP) model, and equivalent uniform dose (EUD). The other is to build statistical models utilizing all the information that is relevant to disease prognosis such as demographics, laboratory tests, images, and dosimetry, to find the relationship between those factors and clinical outcomes. The more data is used, the more accurate the prediction would be. In this regard, artificial intelligence especially machine learning (ML) has a great capacity to process huge and complex data and thus has been used in many areas including medicine. Recently, ML has been introduced into radiation oncology to predict clinical outcomes (Kang et al., 2015; Luo et al., 2020).

Cervical cancer is the third most common cancer and a leading cause to death for women worldwide (Ferlay et al., 2019; Rebecca, 2020). It is one of a few cancers that were first treated with radiation therapy successfully (Mazeron and Gerbaulet, 1998). The treatment of cervical cancer is also one of the most complex and challenging cancer management tasks and may involve all three cancer treatment modalities (surgery, chemotherapy, and radiation therapy (RT)) and all radiation therapy techniques (external beam radiation therapy (EBRT), intracavitary/interstitial brachytherapy (BT), high dose rate (HDR)/low dose rate (LDR) brachytherapy, and permanent seed implant). This paper does not intend to provide a comprehensive review of cervical cancer outcome predictions, but mainly focuses on the prediction results with different methods, the efficacy and limitations of prediction associated with radiation therapy.

### Reported Clinical Outcomes

Actual clinical outcomes are directly derived from the results obtained following up patients. Numerus studies have revealed cervical cancer survival rates for different International Federation of Gynecology and Obstetrics (FIGO) stages (I–IV) and different treatment techniques. In the United States, the 5-year survival rates of cervical cancer patients ranged from 17 to 92% with the all-stage rate of 66% according to the American Cancer Society (American Cancer Society, 2020). Surgery, chemotherapy, and radiation therapy are the treatment options for cervical cancer. The clinical outcomes are associated with treatment modalities and FIGO stages. The actual 5-year survival rates have been reported and are summarized in Table 1 (Brunschwig, 1968; Kim et al., 1988; Landoni et al., 1997; Joslin et al., 2001; Eifel et al., 2004). Severe complications were also reported for 9% of patients with radiation therapy alone (Podczaski et al., 1990) and 20% of patients with chemoradiotherapy (Small et al., 2011). Those reported results were summaries of previous clinical data, but not predictions of clinical outcomes. Mathematical models can establish quantitative relationship between disease-related factors and outcomes and thus predict clinical outcomes based on identified prognostic factors or predictors.

**TABLE 1:** The reported actual clinical outcomes.

Modality	Study	5-year survival rate			
		I	II	III	IV
RT	Joslin et al. (2001)	94.5	62.6	37.3	
	Kim et al. (1988)	83.2	68.9	30.9	27
	Landoni et al. (1997)	84			
Surgery	Brunschwig. (1968)	77.4	51.6		
	Landoni et al. (1997)	88			
RT + surgery	Landoni et al. (1997)	78			
Chemoradiotherapy	Eifel et al. (2004)	81.8		62.6	

### Outcome Prediction Using Conventional Statistical Models

Statistical models have been commonly used to analyze clinical results and also for cervical cancer outcome prediction. To make accurate and meaningful predictions, identifying predictors is critical. The linear regression model was introduced to analyze the correlation between the mRNA expression of Homeobox (HOX) genes in cervical cancer and overall survival. It was found that high HOX expression significantly reduced the overall survival in a cohort of 308 cervical cancer patients and the difference in 15-years survival rate between high and low expression was up to around 25% (Eoh et al., 2017). The Cox proportional hazards regression model (CPHR) uses hazard ratio to distinguish different groups and evaluates the relative importance of predictors. Tumor diameter has been identified as an important predictor based on CPHR (Landoni et al., 1997). A retrospective study reviewed the hospital records of 4,490 patients with stage IB, IIA, or IIB cervical cancer at a single institution, and found that the disease-specific survival (DSS) rate and pelvic disease control (PDC) rate had strong correlations with tumor diameter, FIGO stage, histological subtype, and clinical node status. Overall, the 5-year DSS for tumor diameter ≤4, 4.1–6, and >6 cm, was 85, 69, and 52%, respectively; for stages I, IIA, and IIB disease DSS was 80, 68, and 59%, respectively, and the PDC rates were 90, 87, and 82%, respectively (Eifel et al., 2009).

### Outcome Prediction Using Image Analysis

Radiation therapy heavily relies on medical imaging. Various three-dimensional (3D) imaging techniques such as computerized tomography (CT), nuclear magnetic resonance imaging (MRI) and positron emission tomography (PET) have been widely used for cervical cancer diagnosis and treatment. Those images may also contain the information about clinical outcomes. By analyzing the F-18 fluorodeoxyglucose (FDG) pretreatment images of 248 cervical cancer patients staged from IA2 to IVB and using CPHR, a study reported that the maximal standardized uptake value (SUVmax) that quantifies cervical tumor uptake of FDG is associated with treatment response and prognosis in cervical cancer patients and gave better outcome prediction than lymph node status, stage, or tumor volume (Kidd et al., 2007). The results showed that the overall survival rate at 5 years was 95% for patients with an SUVmax ≤5.2, 70% for patients with an SUVmax from >5.2 to ≤13.3, and 44% for patients with an SUVmax >13.3.

Recently, radiomics has been introduced as a powerful tool to extract huge and complex image features from PET/CT and MRI images for prediction of cervical cancer clinical outcome. It was reported that radiomics features could contribute to prognoses in cervical cancer (Lucia et al., 2018). Using CPHR, two textural features, Grey Level Non Uniformity gray-level run-length matrix (GLRLM) in PET and Entropy gray-level co-occurrence matrix GLCM in ADC maps from DWI MRI, were identified as independent prognostic factors. They were significantly stronger correlated with prognoses than clinical parameters*,* with an accuracy of 94% for predicting recurrence and 100% for predicting lack of loco-regional control compared with ∼50–60% accuracy with clinical parameters. It was also found that the high gray-level run emphasis (HGRE) derived from GLRLM and used to measure high SUV distribution can serve as a predictor (Chen et al., 2018a). This study included 142 cervical cancer patients who had took 18F-FDG PET/CT for pretreatment staging and treated with EBT and intracavitary brachytherapy as well as concurrent chemotherapy. The binary logistic regression model was used to identify the independent prognostic factors among all the radiomic features and predict clinical outcomes. The log-rank test and CPHR analysis were performed to examine the effects of explanatory variables on outcome endpoints including overall survival, progression-free survival, distant metastasis-free survival, and pelvic relapse-free survival. The results showed that the value of HGRE >3.68 or <3.68 were associated with significant different progression-free survival and pelvic relapse-free survival. Thus, HGRE was identified as an important factor in predicting chemoradiotherapy outcomes.

### Outcome Prediction Using Machine Learning

To the author’s knowledge, ML was first used to predict overall survival for 134 cervical cancer patients in 2002, using an artificial neural network model (ANN) including 11 prognostic factors (age, performance status, hemoglobin, total protein in serum, FIGO stage, histological type, histological grading at 30 Gy, histological grading at 40 Gy, histological grading at the end of therapy, cytological grading at 30 Gy, cytological grading at 40 Gy, cytological grading at the end of therapy) (Ochi et al., 2002). The predicted survival result was able to achieve an area under the receiver operating characteristic (ROC) curve (AUC) of 0.7782. A more recent study included 102 patients with cervical cancer staged as IA2-IIB, selected 23 demographic and tumor-related parameters, and collected perioperative data of each patient (Obrzut et al., 2017). The study predicted the 5-year survival rate using six machine learning methods: the probabilistic neural network (PNN), multilayer perceptron network (MLP), gene expression programming classifier (GEP), support vector machines algorithm (SVM), radial basis function neural network (RBFNN) and k-Means algorithm. Compared with other models, PNN provided the best prediction with an accuracy of 0.892 and sensitivity of 0.975. PNN was further used to predict the 10-year survival for the same cohort and also achieved high predictability (Obrzut et al., 2019).

Deep-learning (DL) has also been introduced for outcome prediction. A neural network model was implemented to predict survival utilizing clinicolaboratory variables among recurrent cervical cancer patients (Matsuo et al., 2017). The study tried to find among 13 clinicolaboratory variables the predictors for life expectancy in 157 recurrent cervical cancer patients. Those variables included age, body habitus change, pain score, blood pressure, and heart rate, white blood cell, hemoglobin, platelet, bicarbonate, blood urea nitrogen, creatinine, and albumin. The results showed that the 3-month survival decrease was associated with older age, decreasing albumin level, decreasing body mass index, increasing pain score, decreasing systolic blood pressure, decreasing white blood cell count, increasing platelet counts, and decreasing hemoglobin levels. This study group further predicted survival rate for 768 cervical cancer patients using the same DL model with 40 features that included patient demographics, vital signs, laboratory test results, tumor characteristics, and treatment types (Matsuo et al., 2019). They showed that the results of DL were better than that of CPHR.

In a recent study, a DL model called network in network was developed to predict treatment failures including local relapse and distant metastasis based on the analysis of the PET/CT images (Shen et al., 2019). The prediction of local relapse and distant metastasis obtained reasonable accuracy, sensitivity, and specificity. (Table 2) Four groups of radiomic features were also calculated, but none of the radiomic features was able to predict distant metastasis in this study.

**TABLE 2:** The results from machine learning.

Algorithm	Study	Accuracy	Sensitivity	Specificity	AUC	End point
PNN	Obrzut et al. (2017)	0.9	1.0			5 year-survival
	Obrzut et al. (2019)		0.9	0.7		10-Survival
Network in network	Shen et al. (2019)	89.0	71.0	93.0		Recurrence
		87.0	77.0	90.0		Metastasis
CNN	Zhen et al. (2017)		72.0	59.0	0.700	Rectal toxicity
SVM	Chen et al. (2018b)		87.8	79.9	0.910	Rectal toxicity
SVM	Tian et al. (2019)		97.1	88.5	0.904	Radiation-induced fistula

ML is also able to predict treatment complications. A retrospective study applied the convolutional neural network (CNN) algorithm to analyze rectum dose distribution and predict rectum complications (Zhen et al., 2017). The study included 42 cervical cancer patients treated with EBRT combined with BT. The results showed that the texture features derived from the rectum surface dose map can generate better predictive performance than the volume parameters D_0.1/1/2cc_ that are prescribed for dose constrains, in terms of sensitivity, specificity and AUC. The same research group applied the SVM algorithm to predict rectal toxicity for the same patient cohort and also achieved higher sensitivity, specificity, and AUC when compared with D_0.1/1/2cc_ (Table 2) (Chen et al., 2018b).

The radiation-induced fistula is a concern for treating advanced gynecological (GYN) malignancies using radiation therapy. Another SVM model was developed to predict the risk of fistula formation caused by radiation therapy (Tian et al., 2019). The study included 35 gynecological cancer patients treated with interstitial BT. The model used the features of mixed data types that might be correlated to fistula formation, and included patient demographics, patient health status, tumor characteristics, additional invasive procedures, and dosimetric parameters. The predicted outcomes achieved a high prediction accuracy as shown in Table 2.

## Discussion

Accurate prediction of clinical outcomes would guide treatment to focus on specific prognostic factors and optimize the treatment scheme for each patient. The prediction of cervical cancer outcomes is one of the most challenging tasks as the management of cervical cancer involves the most complicated cancer treatment strategies. The studies reviewed in this paper have utilized models to discover many new prognostic factors such as tumor diameter, histological subtype, FDG SUVmax, radiomic features, and clinicolaboritory variables, and establish the relationships between those factors and clinical outcomes. Therefore, clinical outcomes can be accurately predicted. But the accuracy of prediction is related to models and algorithms. Several models performed very well in the studies. For example, CHPR predicted the 5-year survival rates 80% (I), 68% (IIA), and 59% (IIB) (Eifel et al., 2009), which were comparable to the reported results of 83.2% (I) and 68.9% (II) (Kim et al., 1988). Also, several DL models gave high accuracy predictions (Table 2). Such promising results have indicated that model-based outcome prediction has great potential for clinical applications.

The models used for prediction can be categorized into conventional statistical models and ML models. Conventional statistical models include the linear regression, the logistic regression, and CPHR. CPHR is one of most commonly used models for outcome prediction. It models relative hazards treating all the relevant factors proportionally. It can determine which factor is the most influential. But the proposed proportionality or linearity may not be valid because many prognostic factors are not linear and interact with each other. Thus the performance of prediction may not be ideal. In contrast, ML is able to deal with complex and non-linear relations in the data. Especially, it is able to learn feature representations automatically from raw data without direct feature engineering. Overall, ML outperformed statistical models in cervical cancer outcome prediction (Matsuo et al., 2017; Luo et al., 2019; Matsuo et al., 2019; Tian et al., 2019).

However, there was also evidence that the superiority of ML in outcome prediction is not always supported (Christodoulou et al., 2019). In addition, the ML models and algorithms have their own limitations, notably, overfitting (Zhen et al., 2017), and lack of interpretability (Luo et al., 2019; Luo et al., 2020). Overfitting would undermine predictive performance. Lack of interpretability would hinder the use of ML. ML works like a “black box” due to the complex algorithms. It is not easy to understand how it works and the predicted outcomes are not easy to understand as well. For instance, some predictors such as Albumin level were identified as significant prognostic factors by CPHR, but not by the DL (Matsuo et al., 2017). Thus, the prediction using ML may not be as convincing or well accepted as that using conventional models that are explicitly formulated. Furthermore, it is difficult to catch bugs or errors if they occur. Development of independent validation methods may help resolve this issue.

It should also be realized that the studies reviewed in this paper have limitations as well. First of all, most prediction studies did not have enough data, which would reduce the accuracy of the predictions. Secondly, most studies did not distinguish between treatment modalities and techniques. The treatment of cervical cancer involves almost all available cancer treatment modalities and techniques. Each modality and technique play specific roles and has different contributions to clinical outcomes. For example, LDR brachytherapy led to the 4-year disease-free survivals of 87, 66, and 28% for FIGO stages I, II, and III, respectively, (Coia et al., 1990), while HDR was able to achieve the 5-year survival of 94.4, 62, and 37.2%, for state I, II, III, respectively (Utley et al., 1984). Thus, the impact of different techniques on the outcomes should be determined separately and weighted in the prediction models. More attention should be paid to brachytherapy as brachytherapy is a major and complex treatment modality for cervical cancer. Especially, brachytherapy is sensitive to radiobiological effect. Radiobiological effect such as, dose-rate effect should be included in prediction models. Finally, most studies were limited to a single institution and small number of patients, and the results may have bias and significant uncertainties. The predicted outcomes are expected to be comparable to the actual outcomes independently derived from clinical trials or actual patient records.

## Conclusion

The prediction of cervical cancer outcomes utilizing statistical models, images, and ML has produced promising results. Particularly, ML has capacity to process complex and non-linear relations in large-scale data, discover new prognostic factors, and perform predictions. It has great potential in clinical applications. However, more work is needed to make ML practical and reliable for clinical use. Future studies may include development of new methods and algorithms to minimize the effect of data scarcity, differentiating treatment modalities and techniques in prediction and evaluating individual contributions to clinical outcomes, and independent validation of machine learning algorithms.
